# Calcitonin receptor, calcitonin gene-related peptide and amylin distribution in C1/2 dorsal root ganglia

**DOI:** 10.1186/s10194-024-01744-z

**Published:** 2024-03-14

**Authors:** Tayla A. Rees, Zoe Tasma, Michael L. Garelja, Simon J. O’Carroll, Christopher S. Walker, Debbie L. Hay

**Affiliations:** 1 School of Biological Sciences, University of Auckland, Auckland, 1010 New Zealand; 2 Maurice Wilkins Centre for Molecular Biodiscovery, University of Auckland, Auckland, 1010 New Zealand; 3 Department of Pharmacology and Toxicology, University of Otago, Dunedin, 9016 New Zealand; 4 Department of Anatomy and Medical Imaging, Centre for Brain Research, Faculty of Medical and Health Science, University of Auckland, Auckland, 1023 New Zealand

**Keywords:** Dorsal root ganglia, Amylin, Calcitonin gene-related peptide, AMY receptors, Neuropeptides, Headache, Nociception, G protein-coupled receptors

## Abstract

**Background:**

The upper cervical dorsal root ganglia (DRG) are important for the transmission of sensory information associated with the back of the head and neck, contributing to head pain. Calcitonin receptor (CTR)-based receptors, such as the amylin 1 (AMY_1_) receptor, and ligands, calcitonin gene-related peptide (CGRP) and amylin, have been linked to migraine and pain. However, the contribution of this system to nociception involving the cervical DRG is unclear. Therefore, this study aimed to determine the relative distribution of the CTR, CGRP, and amylin in upper cervical DRG.

**Methods:**

CTR, CGRP, and amylin immunofluorescence was examined relative to neural markers in C1/2 DRG from male and female mice, rats, and human cases. Immunofluorescence was supported by RNA-fluorescence in situ hybridization examining amylin mRNA distribution in rat DRG.

**Results:**

Amylin immunofluorescence was observed in neuronal soma and fibres. Amylin mRNA (*Iapp*) was also detected. Amylin and CGRP co-expression was observed in 19% (mouse), 17% (rat), and 36% (human) of DRG neurons in distinct vesicle-like neuronal puncta from one another. CTR immunoreactivity was present in DRG neurons, and both peptides produced receptor signalling in primary DRG cell cultures. CTR-positive neurons frequently co-expressed amylin and/or CGRP (66% rat; 84% human), with some sex differences.

**Conclusions:**

Amylin and CGRP could both be local peptide agonists for CTR-based receptors in upper cervical DRG, potentially acting through autocrine and/or paracrine signalling mechanisms to modulate neuron function. Amylin and its receptors could represent novel pain targets.

**Supplementary Information:**

The online version contains supplementary material available at 10.1186/s10194-024-01744-z.

## Background

The upper cervical dorsal root (spinal) ganglia (DRG) house cell bodies of sensory neurons, such as the greater occipital nerve, that innervate the vasculature, musculature, and dura of the neck and lower/back of the head [1]. They transmit information, including nociceptive, from the periphery to the central nervous system [2, 3]. Sensitisation of sensory neurons is a key feature of chronic pain and sensitisation of neurons associated with the upper cervical DRG may contribute to disorders such as migraine, cervicogenic headache, and neuropathic pain [4]. Despite its pathophysiological significance, the molecular mechanisms underlying sensitisation are not fully understood, although neuropeptides are thought to contribute by modulating the activity of specific neuronal subtypes [2]. For example, neuropeptide-rich C-fibre neurons may have a more pronounced role compared to other sensory neuron subtypes, like A-fibre neurons [5, 6]. A comprehensive understanding of the molecular processes involved in the neuromodulation of DRG neurons, including identifying new neuropeptide-receptor signalling partnerships, is imperative to understand pathological sensitisation and the transmission of nociceptive information. Such receptor systems may present viable targets for therapeutic intervention, as exemplified by the emergence of a new class of migraine therapeutics targeting the calcitonin gene-related peptide (CGRP) axis [7].

CGRP is a prominent neuropeptide in sensory ganglia, being expressed in approximately 30–60% of neurons [8]. CGRP induces neuronal hyperexcitability, increased firing rates, and upregulation of genes and signalling molecules that promote sensitisation; CGRP is thus heavily implicated in the development of chronic sensory disorders, such as migraine [9–11]. A close relative of CGRP is amylin. Amylin is primarily recognised as a neuroendocrine hormone that is secreted from the pancreas in response to food intake [12]. However, growing evidence also implicates amylin in pain signalling. This includes the expression of amylin-responsive receptor subunits in pain-relevant structures, including C-fibre neurons of trigeminal ganglia (TG), and the induction of nociceptive behaviour and neuronal activation in response to peripheral amylin administration, similar to CGRP [13–21]. In addition, exogenous amylin administration can produce anti-nociceptive effects, indicating that its role in pain is complex [19, 22, 23]. Therefore, it is unclear whether endogenous amylin modulates sensory processing or whether its effects are limited to exogenous administration of amylin receptor agonists.

Critically, there is no clear consensus on amylin peptide expression in sensory ganglia. Although some investigation of DRG and TG has occurred, the data are challenging to interpret, for example, due to the cross-reactivity of amylin antibodies with CGRP [16, 17, 20, 21, 24–29]. In addition, the majority of studies tend to examine one sex, focus on lumbar DRG, rather than the migraine-relevant upper cervical DRG, and, to our knowledge, have not examined amylin protein expression in humans [20–22, 24–27, 30]. To determine whether amylin is a physiologically-relevant neuropeptide it is crucial to determine whether amylin is expressed in pain-relevant locations and how any expression relates to that of its key signalling protein, the calcitonin receptor (CTR). This G protein-coupled receptor (GPCR) is the core signalling subunit of several amylin receptor subtypes, some of which can also be potently activated by CGRP [31]. Therefore, understanding CTR expression is also vital from a CGRP perspective, and current research on its expression in the DRG, particularly in humans, is sparse [22, 32]. This study aimed to compare the spatial distribution of amylin relative to CGRP and CTR in the upper cervical (C1/2) DRG of mice, rats, and humans, to investigate the potential of the amylin-CTR axis as a novel neuromodulatory neuropeptide-receptor signalling partnership.

## Methods

### Antibodies and plasmids

All plasmids and antibodies are detailed in Table S1. Three well-validated anti-CTR antibodies were used [13, 14]. This allowed multiple combinations of antibodies in different species to investigate the relative localisation of the proteins of interest.

### HEK293S cell culture and transfection

HEK293S cells were cultured and transfected with plasmids for rat *Iapp*, *Calca*, *Calcb* or empty vector (pcDNA3.1+) as previously described [13, 33].

### Tissue collection for immunofluorescence and RNA fluorescent in situ hybridization

Three male and three female Sprague Dawley (SD) rats and C57BL/6J mice were obtained from the Integrated Physiology Unit (University of Auckland, Auckland, New Zealand). Three additional SD rats (two female, one male) were obtained from the Biomedical Research Facility (University of Otago, Dunedin, New Zealand). Animal ethics, care and euthanasia protocols are detailed in the Supplemental Methods. All procedures involving the use of rodents and their care were approved and conducted in accordance with the ARRIVE2 guidelines [34].

DRG (C1/2) were dissected immediately after euthanasia. For immunofluorescence, DRG were fixed in 4% paraformaldehyde (PFA) for 24 h at 4 ^o^C, cryoprotected with 10% sucrose, then 20% sucrose (W/V) in phosphate-buffered saline (PBS), then embedded in optimal cutting temperature compound (OCT) (Sakura Tissue-Tek, 4583) [17]. For RNA-FISH, DRG were frozen/embedded in OCT immediately after dissection. DRG were sectioned (Leica CM1850 microtome, Leica Biosystems, Wetzlar, Germany) cross-sectionally (12 μm), mounted onto slides and stored at − 80 °C. Post-mortem human DRG ethics and processing are as previously described for human TG and are detailed, alongside case details, in the Supplemental Methods [17].

### Immunofluorescence

Sections were thawed (mouse and rat) or dewaxed and rehydrated (human) as previously described [14, 17]. Antigen retrieval was performed using 10 mM sodium citrate buffer, pH 6.0 for 1 min in the microwave (rodent) or 121 ^o^C for 20 min in an immunohistochemistry pressure cooker (human). Sections were washed twice with tris buffered saline (TBS) + 0.1% tween20 (TBS-T), blocked with 10% normal goat serum (W/V) for 1 h at RT, and then incubated with primary antibodies (Table S1) overnight at 4 ^o^C. Sections were washed twice with TBS-T and incubated with secondary antibodies (1:200, Table S1) and DAPI for 1 h (mouse/rat) or 3 h (human) at RT. Sections were then washed with TBS-T, and coverslips were mounted. β tubulin III was used as a pan-neuronal marker (rodents and humans) and NF200 as an A-fibre neuron marker for rodents but not humans, as NF200 does not discriminate between human neuron types [8]. We are confident that the antibodies used in this study detect their targets, as they have been thoroughly characterised and validated, including using knockout mouse models [13, 14, 28]. However, a number of the antibodies are polyclonal or display a small amount of cross-reactivity for related proteins, i.e. Ab254259 has a small amount of cross-reactivity with rodent, but not human, CGRP [28]. Therefore, we use the descriptions, “immunoreactivity” or “like-immunoreactivity” (LI), to account for this limitation and the potential for some off-target immunoreactivity.

### RNA fluorescent in situ hybridization (FISH)

Fifteen custom probe pairs were generated by Molecular Instruments against the rat *Iapp* mRNA sequence (NM_012586.1). Probes were checked by alignment in Geneious Prime 2020.0.5 (https://www.geneious.com) and BLAST to confirm that they would not bind to rat *Calca* or *Calcb* mRNA or other off-target mRNA sequences. RNA-FISH was performed per the manufacturer’s (Molecular Instruments, Los Angeles, CA) instructions.

Briefly, fresh-frozen rat DRG sections were thawed at RT and fixed with 4% PFA for 15 min. Sections were incubated sequentially with 50%, 75%, and 100% ethanol and then washed with PBS. Sections were incubated with kit hybridization buffer for 10 min at 37 ^o^C, then with probe solution (1.6 µM rat *Iapp* probe in kit hybridization buffer) overnight at 37 ^o^C. Sections were washed sequentially with kit probe wash buffer (PWB) in combination with increasing amounts of saline-sodium citrate buffer + 0.1% triton (SSCT), 100% PWB/0% SSCT, 75% PWB/25% SSCT, 50% PWB/50% SSCT, 25% PWB/75% SSCT, 0% PWB/100% SSCT. Sections were preincubated with amplification buffer for 30 min at RT, then amplification buffer containing hairpins (2 µM) overnight at RT. Sections were washed thrice with SSCT, incubated with DAPI, and coverslips mounted.

HEK293S cells were fixed with 4% PFA for 10–15 min 24–36 h after transfection with *Iapp*, *Calca*, *Calcb*, or empty vector plasmid. RNA-FISH was performed as described for rat DRG sections, with the following adjustments: no ethanol incubations were performed, and the probe concentration was 0.4 µM.

### Fluorescent imaging

Immunofluorescence and RNA-FISH sections and transfected cells were imaged using an Operetta high-content imaging system in non-confocal mode using a 20x high-numerical-aperture (0.8) objective (Perkin Elmer Life and Analytical Sciences, Waltham, MA). Immunofluorescence imaging for CTR, CGRP, and amylin was also performed using a 63x high numerical-aperture (1.15) objective with the Opera Phenix Plus High-Content Screening System in confocal mode (Perkin Elmer Life and Analytical Sciences). Image processing and quantification of the immunofluorescence in mouse, rat and human DRG is described in detail in the Supplemental Methods.

### Generation of primary Wistar rat neonatal DRG cultures and cAMP assays

Three to five-day-old Wistar pups were euthanised by decapitation. Animal ethics, care and euthanasia protocols are detailed in the Supplemental Methods. DRG were dissected, and neurons were enriched and isolated as previously described for neonatal rat TGs [15]. DRG neuron-enriched cells were plated into poly-D-lysine/laminin-coated 384 well cell culture plates (approximately 2 DRG per well) and maintained in a humidified incubator at 37^o^C. After 24 h, LANCE ultra-cAMP signalling assays (PerkinElmer, Waltham, MA) were performed as previously described for neonatal TG cultures [15]. Briefly, cAMP assays were performed with 1 mM 3-isobutyl-1-methylxanthine (IBMX; Sigma-Aldrich, St. Louis, MO). DRG neurons were stimulated with peptides serially diluted in cAMP assay media (L15 medium + 0.1% BSA + 1 mM IBMX) or forskolin (positive control) for 30 min at RT. cAMP content was determined using the LANCE ultra-cAMP detection kit.

### Experimental design and data analysis

In all experiments, the position of the antibodies (immunofluorescence), probes (RNA-FISH), and agonists (signalling assays) were randomised on the slides or 384-well plates between independent experiments. For immunofluorescence and RNA-FISH, independent experiments were defined as individual mice, rats, or human cases, which were treated with independent dilutions of antibodies or RNA-FISH probes/hairpins. For primary DRG culture signalling, independent experiments involved plating of DRG cells from different litters and separate signalling assays. Each independent DRG signalling experiment consisted of two or three technical replicates. Sample sizes for immunofluorescence and signalling assays were based on previous work, where they were found to be sufficient to reach statistical significance [14, 15]. The requirement for multiple distinct antibodies per immunofluorescence condition (triple-staining) and for agonist concentration-response curves to be made up by a single operator for individual imaging or signalling assays resulted in blinding not being feasible. Bias was minimised through our image analysis approaches.

Graphing and statistical analysis were performed using Prism GraphPad 8.0.2 (GraphPad Software, La Jolla, CA). Data shown are the means ± s.e.m from n independent experiments, combined. For image analysis protocols and statistical analysis of this data, see the Supplemental Methods. For primary DRG culture signalling, concentration-response curves were fitted in each individual experiment using three or four-parameter nonlinear regression as determined by F-test. Individual pEC_50_ and E_max_ values were combined to generate mean data and analysed by two-tailed unpaired Student’s *t*-test (rat amylin vs. rat αCGRP). Statistical significance was defined as *p* < 0.05. Data were normalised to the maximum response of rαCGRP in each individual experiment, combined and presented as mean ± s.e.m. from seven (rat amylin) or nine (rat αCGRP) independent experiments. Two CGRP experiments were performed without amylin.

## Results

### Amylin and CGRP are expressed in the DRG

Our first aim was to determine whether amylin is expressed in the DRG. To help interpret this expression, CGRP and neuronal markers were included. β tubulin III was used as a pan-neuronal marker in all species. NF200 was also used in rodent samples as an A-fibre neuron marker, but was not used in human samples because NF200 does not discriminate between human neuron subtypes [8]. We used anti-amylin and anti-CGRP antibodies previously shown to have minimal or no cross-reactivity between each peptide [28]. This is important because amylin and CGRP share approximately 50% amino acid sequence identity as well as structural similarities, meaning that off-target detection is a common confounder in data interpretation [28, 29, 35]. The CGRP antibody used detects αCGRP and βCGRP, therefore, all CGRP immunoreactivity is considered “pan-CGRP” (Figure S1).

**Fig. 1:**
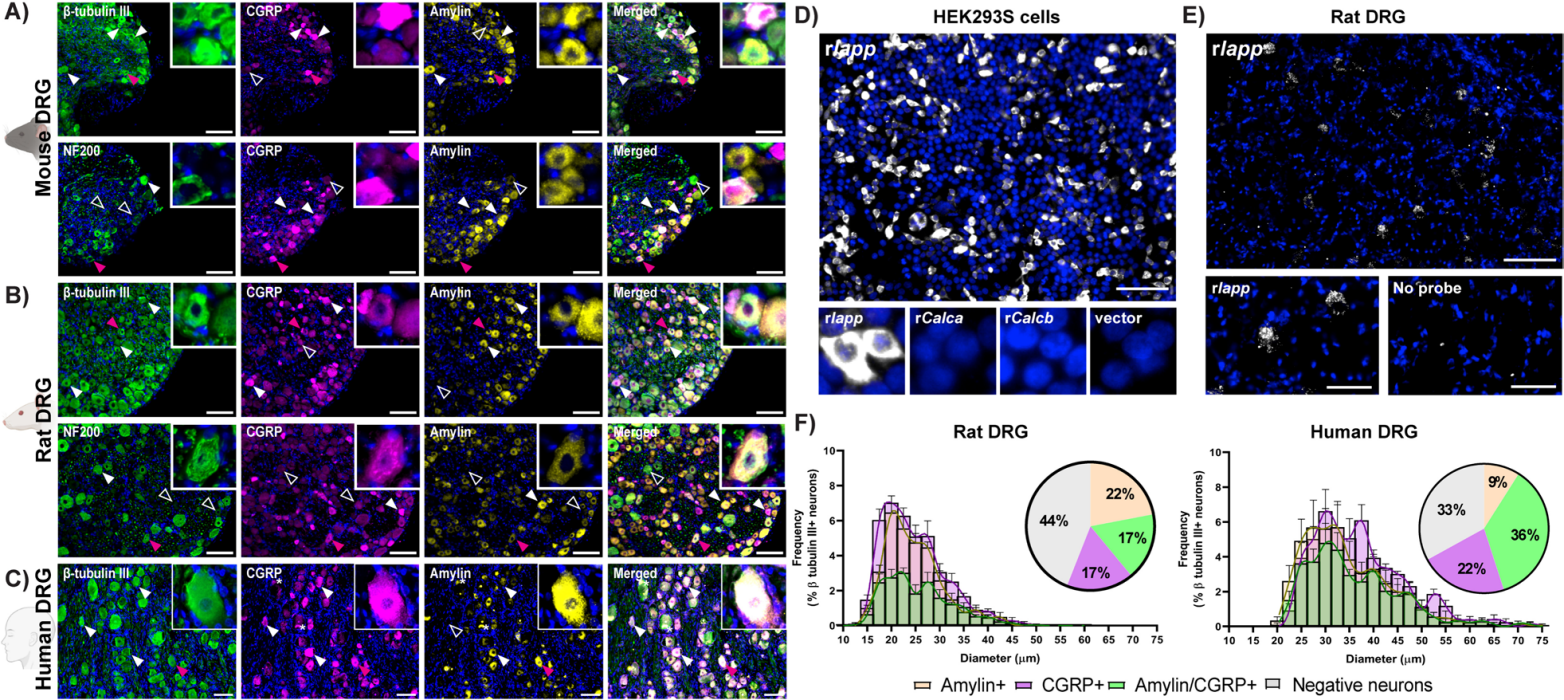
Amylin and CGRP are expressed in DRG neurons. Immunofluorescent localisation of amylin and CGRP with β tubulin III or NF200 for (**A**) mouse, (**B**) rat and (**C**) human DRG using anti-amylin (mAb254259) and anti-CGRP (pAb36001) antibodies. Filled arrowheads indicate examples of positive immunoreactivity; empty arrowheads indicate examples of an absence of immunoreactivity. Magenta arrowheads indicate inset location. Image brightness and contrast were adjusted for presentation purposes and merged in FIJI. Scale bar, 100 μm. Images are representative of six mice or rats (three male, three female) and four human cases (two male, two female). **D** Validation of the rat Iapp RNA-FISH probe in transfected HEK293S cells, images representative of three independent experiments. **E** RNA-FISH detection of rat *Iapp* mRNA in the presence or absence of 1.6 µM rat *Iapp* probe in rat C1 DRG. Fluorescent in situ hybridization shown in greyscale and nuclear staining in blue. Scale bar, (**D**, **E**) 100 μm or (E, insets) 50 μm. Images are representative of results from three individual rats (two male, one female). **F** The distribution of neuron size quantified relative to the total β tubulin III expressing neuron population and the percentage of total rat and human DRG neuronal population (β tubulin III) which express amylin alone, CGRP alone, or co-express amylin and CGRP together (immunohistochemistry only). Negative neurons refers to the population of neurons (β tubulin III-positive cells) which do not express amylin or CGRP. Size distribution of the mouse DRG neurons could not be performed due to limitations with the image analysis

Immunoreactivity for CGRP and amylin were present in DRG cell bodies of mice, rats and humans (Fig. 1A, B, C). There was variation in CGRP and amylin immunoreactivity between the human cases (Figure S2). Immunoreactivity for both peptides was sometimes present in neuronal cell bodies, which expressed high levels of NF200 (Fig. 1A, B). Given literature inconsistencies regarding amylin expression in sensory ganglia [16, 24, 28], we substantiated our amylin expression finding with an alternative approach, by examining amylin (*Iapp*) mRNA using RNA-FISH. Probe validation showed that the *Iapp* probe detected rat *Iapp* but did not cross-react with rat αCGRP (*Calca*) or βCGRP (*Calcb*) mRNA in transfected cells (Fig. 1D). In rat DRG, fluorescent puncta were observed, indicating rat *Iapp* mRNA expression (Fig. 1E).

To gain additional insights from our immunoreactivity data, we used image analysis to quantify and compare the expression patterns of CGRP and amylin, relative to the pan-neuronal marker β tubulin III in the DRG. Examining the data set as a whole across species and sex, at least 51% of β tubulin III-positive DRG neurons expressed one or more of these peptides (Fig. 1F; Table 1). The presence of amylin but not CGRP in some cell bodies supports this being genuine amylin immunoreactivity. The lower and upper range of the total percentage of neuronal cell bodies expressing amylin was 33–54%. This was 31–62% for CGRP (Table 1). The lower and upper range of β tubulin III-positive neurons that were immunoreactive for both amylin and CGRP was 16–39% (Table 1). In rats, we were also able to quantify what percentage of peptide immunopositive neurons also expressed high levels of the A-fibre marker NF200 (Table 2). The percentage of CGRP, amylin, and CGRP and amylin together in immunopositive neurons which also expressed NF200 was 52%, 38%, 53%, respectively (Table 2). The statistical comparisons between peptides, sex and species are provided in the final results section, to enable comparison with CTR.

**Table 1:** Percentage of neurons (β tubulin III) that express CGRP, amylin, CGRP and amylin together, CTR, and CTR and CGRP together in mouse, rat, and human DRG.

Species	Sex		% β tubulin III positive neurons				
			CGRP	Amylin	CGRP/amylin	CTR	CTR/CGRP
**Mouse**	Combined (6)	**Total**	32 ± 2.1*	38 ± 4.0	19 ± 1.4*	44 ± 3.9	27 ± 4.2
		**Only**	13 ± 1.2	19 ± 3.0		17 ± 3.2	
	Female (3)	**Total**	33 ± 2.9	43 ± 5.1	20 ± 2.6	39 ± 4.1	26 ± 6.0
		**Only**	13 ± 0.4	23 ± 2.9		14 ± 6.3	
	Male (3)	**Total**	31 ± 3.6	33 ± 5.3	18 ± 1.4	49 ± 6.3	29 ± 7.0
		**Only**	13 ± 2.6	15 ± 4.4		20 ± 0.7	
**Rat**	Combined (6)	**Total**	34 ± 2.3*	39 ± 1.9	17 ± 1.8*	43 ± 2.5	23 ± 3.2
		**Only**	17 ± 1.7	22 ± 1.9*		20 ± 1.2	
	Female (3)	**Total**	31 ± 2.4	38 ± 3.9	16 ± 3.2	42 ± 4.8	21 ± 6.4
		**Only**	15 ± 3.1	22 ± 3.5		21 ± 1.8	
	Male (3)	**Total**	36 ± 3.7	39 ± 1.8	18 ± 2.3	44 ± 2.4	25 ± 2.3
		**Only**	18 ± 1.3	21 ± 2.3		19 ± 1.8	
**Human**	Combined (4)	**Total**	58 ± 6.5	45 ± 11	36 ± 7.0	47 ± 4.7	32 ± 7.4
		**Only**	22 ± 5.5	9 ± 4.5		15 ± 2.8	
	Female (2)	**Total**	53 ± 14	54 ± 21	39 ± 13	41 ± 7.0	21 ± 9.2
		**Only**	14 ± 0.7	15 ± 7.1		20 ± 2.2	
	Male (2)	**Total**	62 ± 2.5	36 ± 11	33 ± 10	54 ± 1.6	43 ± 1.6
		**Only**	29 ± 7.1	3 ± 1.9		11 + 0.0	

The “total” number represents the total percentage of β tubulin III neurons which express CGRP, amylin, CGRP and amylin together, CTR, or CTR and CGRP together. CGRP “Only” describes the percentage of β tubulin III neurons which express CGRP but not amylin. Amylin “only” describes the percentage of β tubulin III neurons which express amylin but not CGRP. CTR “ only” describes the percentage of β tubulin III neurons which express CTR but not CGRP. Combined data are the mean ± s.e.m from six individual rats or mice (three female and three male) or four human cases (2 female and 2 male), individual animal or case numbers are shown as (n)

*P < 0.05 by one-way ANOVA with post-hoc Bonferroni’s test comparing the total or only “Combined” % β tubulin III positive neurons expressing CGRP, amylin, CGRP/amylin, CTR or CTR/CGRP for mice or rats to humans. Females and males within each species for each condition were compared by unpaired Students’ *t*-test; no significant differences were observed.

**Table 2:** Percentage of CGRP, amylin, CGRP and amylin co-expressing, CTR, and CTR and CGRP co-expressing, positive neurons that co-express NF200 (A-fibre marker) in rat DRG

Species	Sex	CGRP total	Amylin total	CGRP/amylin total	CTR total	CTR/CGRP total
**Rat**	Combined (6)	52 ± 4.2	38 ± 5.0	53 ± 5.3	28 ± 2.3	35 ± 2.9
	Female^a^ (3)	46 ± 6.1	29 ± 1.0	44 ± 5.8	26 ± 0.8	31 ± 2.7
	Male^a^ (3)	58 ± 3.6	46 ± 6.8	**63 ± 3.5^**	31 ± 4.4	39 ± 4.2

Data represent the percentage of CGRP, amylin, CGRP and amylin, CTR, or CTR and CGRP expressing neurons that also express NF200. Combined data are the mean ± s.e.m from six individual rats (three female and three male, individual animal numbers are shown as (n)

^*P* < 0.05 by unpaired Students’ *t*-test comparing between females and males within each species for each condition.

^a^There are no significant differences in the total number of NF200 or β tubulin III expressing neurons between female and male rats by unpaired Students’ *t*-test. Analysis of percentage of peptide and receptor co-expressing NF200 could not be performed in humans and mice, due to NF200 being unable to discriminate between human neuron subtypes (human) and constraints with the image analysis (mice, detailed in the Supplemental methods)

Figure 1F shows the size distribution of β tubulin III-positive rat and human DRG neurons that were immunoreactive for amylin and/or CGRP. In rats, amylin and/or CGRP were largely found in neurons 15-50 µm in diameter, being most abundant in neurons approximately 17.5-35 µm in diameter (Fig. 1F). In humans, neuron size increased with amylin and/or CGRP being present in neurons 20-75 µm in diameter, with the greatest abundance in neurons of the 22.5-50 µm in diameter range (Fig. 1F).

### The CTR is expressed in the DRG

**Fig. 2:**
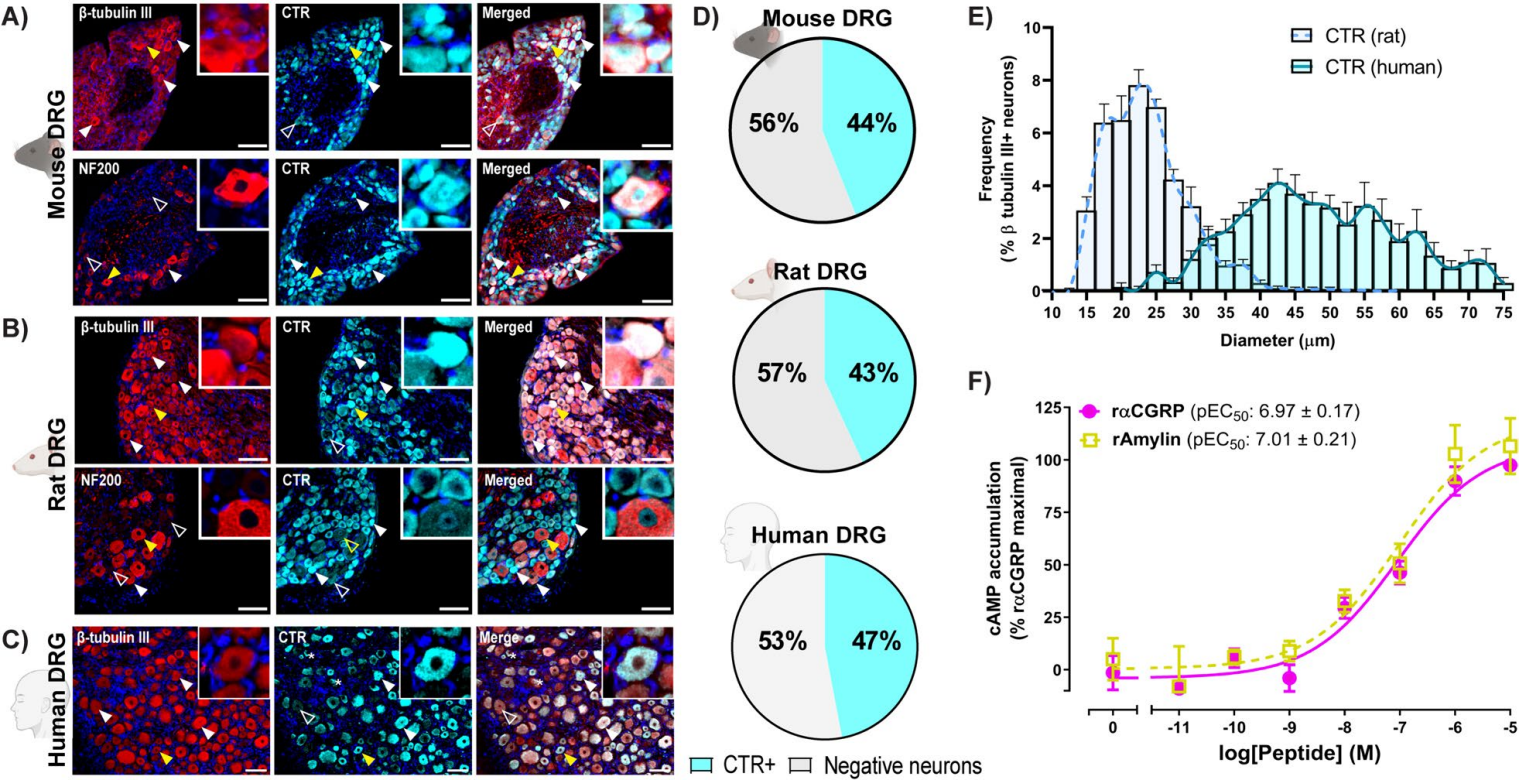
CTR is expressed in DRG neurons. Immunofluorescent localisation of CTR with β tubulin III or NF200 in (**A**) mouse, **B** rat, and (**C**) human DRG using anti-CTR (mouse/rat: pAb188/10; human: mAb31-01/1H-10) antibodies. Filled arrowheads indicate examples of positive immunoreactivity; empty arrowheads indicate examples of an absence of immunoreactivity. Yellow arrowheads indicate inset location. Image brightness and contrast were adjusted for presentation purposes and merged in FIJI. Scale bar, 100 μm. Images are representative of six mice or rats (three male, three female) and four human cases (two male, two female). **D** The percentage of total DRG neuronal population (β tubulin III-positive cells) which express CTR. Negative neurons refers to the population of neurons (β tubulin III-positive cells) which do not express CTR. **E **The distribution of neuron size quantified relative to the total β tubulin III expressing neuron population. **F** cAMP production in response to rat αCGRP and amylin in neuron-enriched neonatal rat DRG cultures. Data points are mean ± s.e.m, combined from 7–9 independent experiments performed in duplicate or triplicate

We next explored whether CTR, a shared GPCR for both amylin and CGRP, was also expressed in the DRG and therefore could form a local signalling hub. CTR immunoreactivity was observed in β tubulin III-positive neuronal cell bodies of all species (Fig. 2). The intensity of this immunoreactivity varied between cell bodies, sometimes being intense, and sometimes diffuse and granular (Fig. 2A-C). When quantified, 39–54% of β tubulin III-positive DRG neurons were immunoreactive for CTR (Fig. 2D; Table 1). There was variation in CTR immunoreactivity between the human cases (Figure S3).

CTR immunoreactivity was occasionally present in neurons that had high levels of NF200 expression (Fig. 2A-B). This could be quantified in rat samples, and accounted for 26–31% of all CTR-positive cells (Table 2). Interestingly, the size distribution of CTR-positive neurons was different between rats and humans (Fig. 2E). In rats, the distribution was narrow with most CTR being observed in neurons approximately 17.5–30 μm in diameter, suggesting expression in small to medium-sized neurons [36]. The size distribution in human DRG samples was broader, with CTR immunoreactivity observed in neurons from 25 to 75 μm in diameter (Fig. 2E).

To determine whether this CTR immunoreactivity could be functionally important, primary DRG neuronal cultures from neonatal rats were prepared and stimulated with rat αCGRP or rat amylin before being assayed for cAMP as a measure of intracellular signalling in response to receptor activation (Fig. 2F). Concentration-dependent increases in cAMP accumulation were observed in response to both peptides. There were no significant differences between peptide potencies (αCGRP pEC_50_, 6.97 ± 0.17, *n* = 9; amylin pEC_50_, 7.01 ± 0.21, *n* = 7) or maximal response (αCGRP E_max_, 3.78 ± 0.74 nM, *n* = 9; amylin E_max_, 3.25 ± 0.49 nM, *n* = 7). These relative potencies are consistent with a potential rodent CTR-based receptor [37, 38].

### The CTR, CGRP and amylin are co-expressed in the DRG

**Fig. 3:**
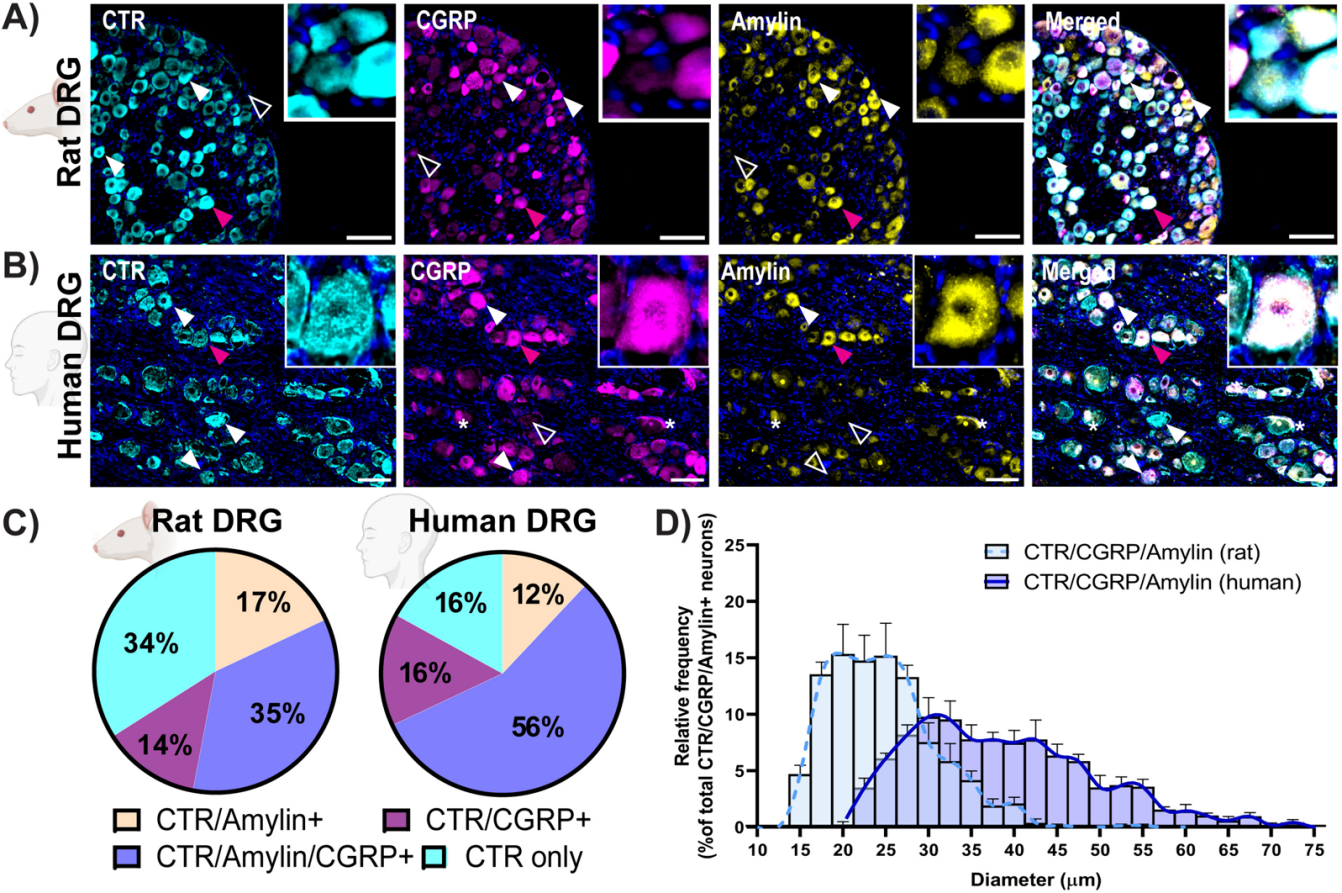
CTR, CGRP and amylin are expressed in DRG neurons. Immunofluorescent localisation of CTR, CGRP and amylin in (**A**) rat or (**B**) human DRG using anti-amylin (mAb254259), anti-CGRP (pAb36001) and anti-CTR (rat: mAb8B9; human: mAb31-01/1H-10). Filled arrowheads indicate examples of positive immunoreactivity; empty arrowheads indicate examples of an absence of immunoreactivity. Magenta arrowheads indicate inset location. Image brightness and contrast were adjusted for presentation purposes and merged in FIJI. Scale bar, 100 μm. Images are representative of six rats (three male, three female) or four human cases (two male, two female). **C** The percentage of CTR-positive DRG neurons that also co-express amylin, CGRP, or amylin and CGRP together. CTR only neurons refers to the population of CTR-positive neurons which do not express amylin or CGRP. **D **The distribution of size of neurons co-expressing CTR, CGRP and amylin together was quantified relative to the number of neurons which co-express CTR/CGRP/amylin for rat and human DRG

To determine whether the receptor and peptides were expressed in the same or distinct neuronal populations we compared their relative spatial distribution in rat and human DRG sections by co-incubating with antibodies against all three targets. To use three primary antibodies raised in different species in rat samples we substituted the CTR antibody 188 with the CTR antibody 8B9 in these experiments. As 8B9 is a mouse monoclonal antibody, the same three-way comparison could not be done in mouse samples. Different populations of neuronal cell bodies were evident, with different combinations of immunoreactivity. CTR could be found together with CGRP or amylin, or both peptides together in both species (Fig. 3A, B). Occasionally, peptides but not CTR were present, though CTR could be found near a peptide-positive cell (Fig. 3A, B). Cell bodies with only CTR immunoreactivity were also evident. Quantification is shown in Fig. 3C and D; Table 3.

**Table 3:** Percentage of CTR positive neurons which co-express CGRP alone, amylin alone or CGRP and amylin together in rat and human DRG

Species	Sex	% CTR positive neurons			
		CGRP only	Amylin only	CGRP/Amylin	CTR alone
**Rat**	Combined (6)	14 ± 3.7	17 ± 4.7	**35 ± 4.3**^**✝**^	**34 ± 3.2**^**✝**^
	Female (3)	9 ± 2.5	27 ± 2.1	31 ± 7.7	33 ± 4.9
	Male (3)	18 ± 6.6	**7 ± 3.9^**	39 ± 4.2	36 ± 5.1
**Human**	Combined (4)	16 ± 4.6	12 ± 2.7	56 ± 4.5	16 ± 3.2
	Female (2)	14 ± 7.4	16 ± 4.5	54 ± 10	16 ± 7.2
	Male (2)	17 ± 8.3	9 ± 1.5	57 ± 4.0	17 ± 2.8

Combined data are the mean ± s.e.m from six individual rats (three female and three male) or four human cases (2 female and 2 male), individual animal or case numbers are shown as (n)

^*P* < 0.05 by unpaired Students’ *t*-test comparing between females and males within each species for each condition

^✝^*P* < 0.05 by unpaired Students’ *t*-test comparing the “Combined” % CTR-positive neurons expressing CGRP only, amylin only, CGRP and amylin together, or no co-expression with peptide between rats and humans

### Amylin and CGRP are expressed in different vesicles and fibres

**Fig. 4:**
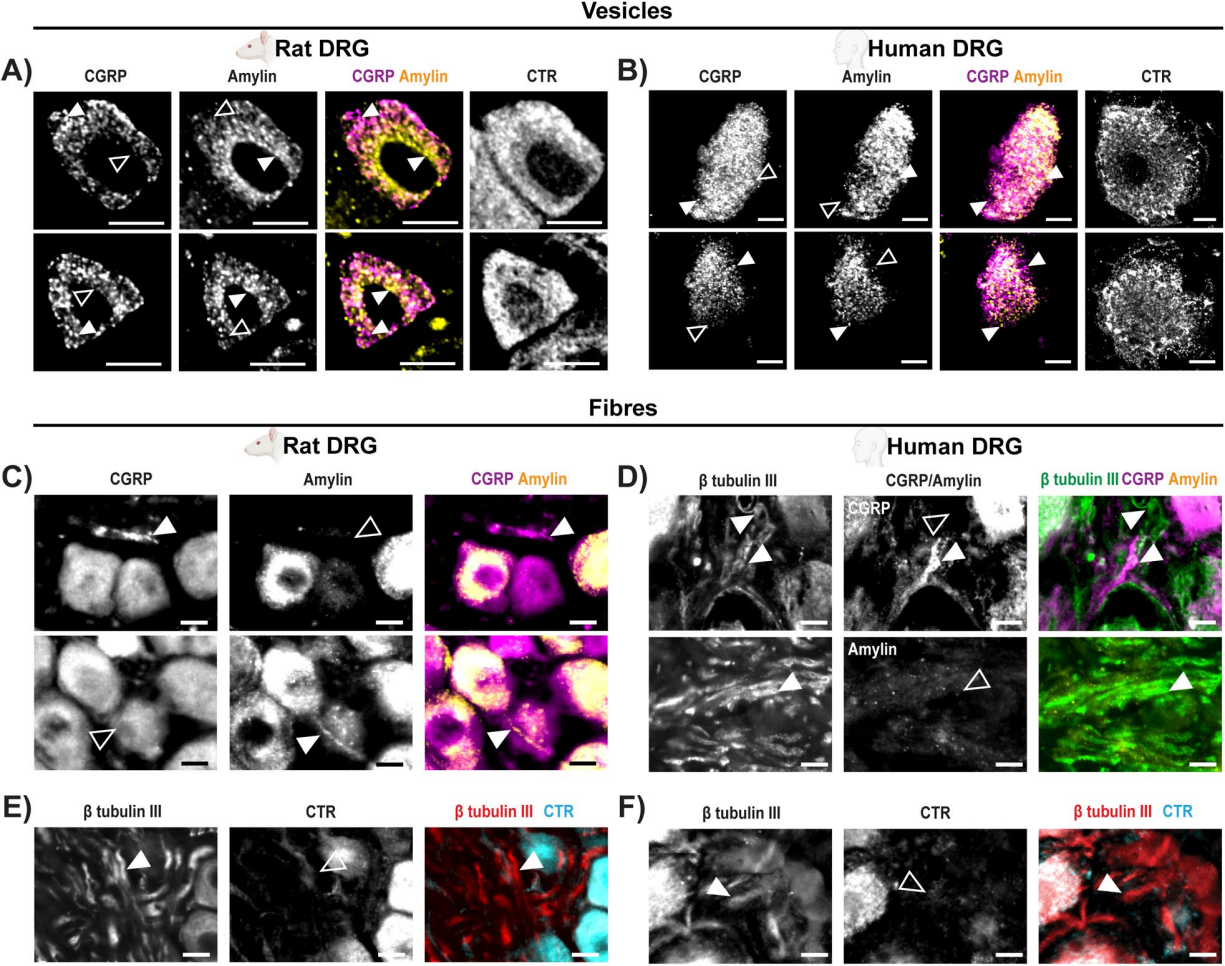
CGRP and amylin are expressed in different vesicles and fibres in the DRG. Antibodies: anti-amylin (mAb254259), anti-CGRP (pAb36001) and anti-CTR (rat: mAb8B9; human: mAb31-01/1H-10). Examples of (**A**) rat and (**B**) human DRG neurons which express both amylin and CGRP. Arrows indicate examples of vesicles which display immunoreactivity for one peptide but not the other. Filled white arrowheads indicate examples of positive immunoreactivity; empty arrowheads indicate examples of an absence of immunoreactivity. **C** Examples of rat DRG fibres that are immunoreactive for one peptide but not the other. **D **Examples of peptide immunoreactivity in human DRG fibres. Examples of (**E**) rat and (**F**) human DRG CTR immunoreactivity relative to neuronal fibres (β tubulin III). Filled white arrowheads indicate examples of positive staining; empty arrowheads indicate examples of an absence of staining. Image brightness and contrast were adjusted for presentation purposes and merged in FIJI. Scale bar, 10 μm. Images are representative of (A) three (one male, two female) or (**C**, **E**) six (three male, three female) rats, or (**B**, **D**, **F**) four human cases (two male, two female). In human DRG, CTR could not be visualised in the same section due to equipment restraints/limitations and secondary antibody combinations

The presence of both peptides in the same neurons provided the opportunity to compare their subcellular localisation using confocal imaging at 63x magnification (Fig. 4A, B). This comparison between CGRP and amylin was performed for rat and human samples; CTR was also included to investigate receptor expression patterns. This work was performed using the Opera Phenix system, which has a different filter set than the Operetta which we used for imaging at 20x magnification. For rat, CTR immunoreactivity could be imaged in the same sections as for CGRP and amylin. For human, CTR immunoreactivity was imaged in adjacent sections because of limitations with microscope filter sets, high autofluorescence for human tissue in the green channel, and antibody combinations that precluded testing of all three on the same section. In neuronal cell bodies, vesicle-like puncta contained only CGRP, only amylin, or both peptides (Fig. 4A, B; Figure S4). CTR immunoreactivity was intense and somewhat granular in rat and more granular with a puncta-like appearance in human (Fig. 4A, B). In rat DRG, neuronal fibres tended to be either CGRP or amylin-positive (Fig. 4C, Figure S5). In human DRG, CGRP was observed occasionally in neuronal fibres (as indicated by β tubulin III) but amylin immunoreactivity was not detectable in fibres under the conditions used (Fig. 4D). No clear CTR immunoreactivity was observed in neuronal fibres of rat or human DRG (Fig. 4E, F).

### There are significant differences in the expression of amylin, CGRP, and CTR between sex, species, and ganglia

**Fig. 5:**
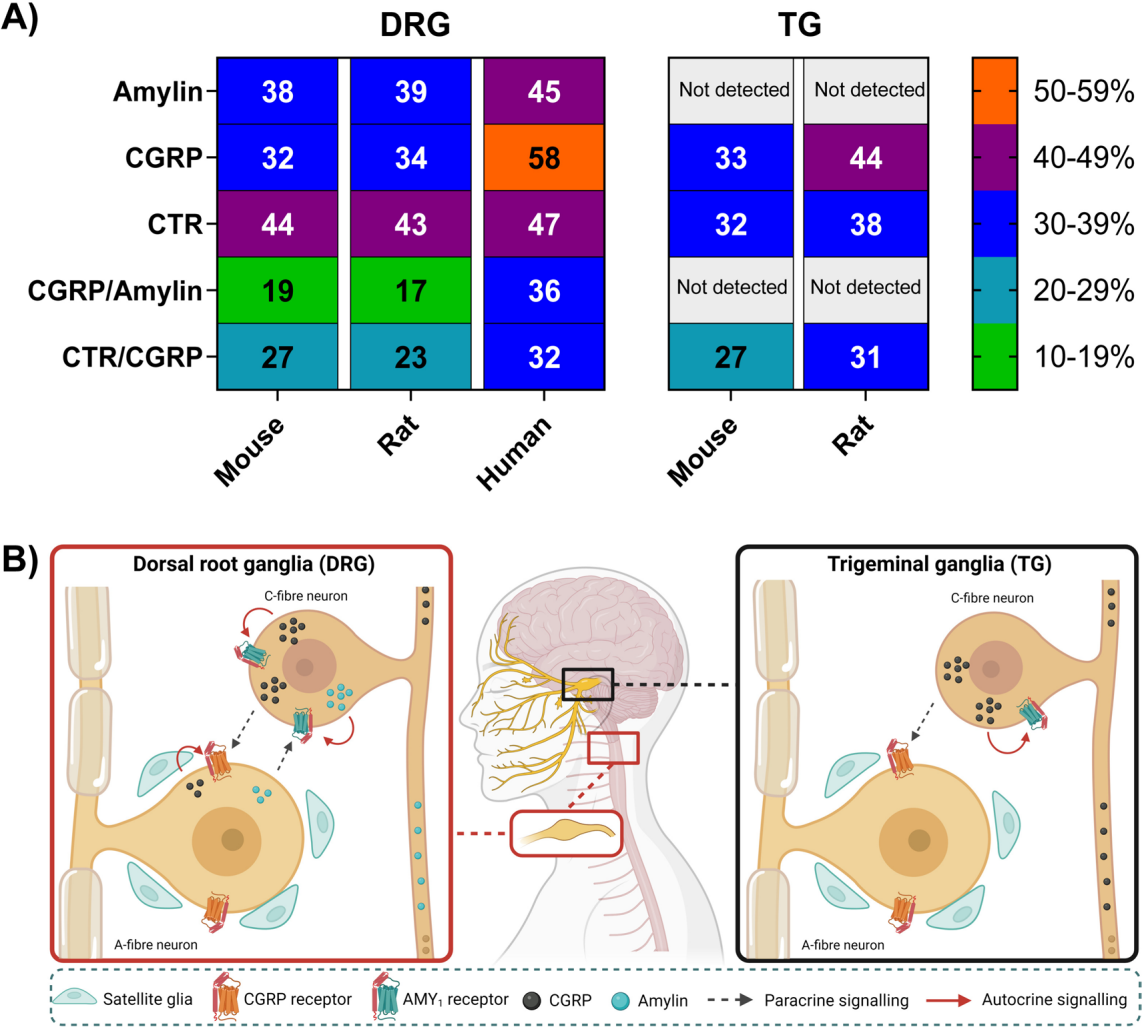
Comparison of the expression of CTR, CGRP and amylin between the C1/2 DRG and TG. **A **Comparison between rat, mouse and human DRG of the total percentage of the DRG neuronal population (β tubulin III) which express CTR, CGRP and amylin alone or together, compared to previous results from rat and mouse TG [14, 17]. Comparisons to the previous human TG data could not be performed due to the variability in human case immunofluorescence. Rat and mouse are characterised as having no detection of amylin in the TG based on the small amount of cross-reactivity of the anti-amylin antibody Ab254259 with rodent CGRP and clear overlap of immunoreactivity with CGRP-LI [17, 28]. **B **Summary diagram comparing the expression patterns of CGRP-responsive receptors, CGRP and amylin in the DRG and TG. These key differences in distribution of migraine and pain-relevant proteins in the TG and DRG may result in distinct mechanistic possibilities for paracrine or autocrine signalling

We first compared peptide expression between species and sex. Human DRG had a significantly greater proportion of β tubulin III positive neurons expressing CGRP and amylin/CGRP than rat and mouse DRG (Fig. 5A; Table 1). There were no significant differences between males and females in the total proportion of neurons (β tubulin III) displaying CGRP, amylin, or both CGRP and amylin immunoreactivity for any species (Table 1). Next, we compared the co-expression of the peptides with NF200 in rats between sexes. Female rats had fewer CGRP-positive, amylin-positive, and CGRP/amylin-positive neurons that co-expressed NF200 than male rats (Table 2). However, this difference was only significant for CGRP/amylin-positive neurons that co-expressed NF200 (Table 2).

The percentages of neurons expressing CTR were compared between species and sex (Fig. 5A; Table 1). No differences were observed. Next, cells co-expressing CGRP, amylin, and CTR immunoreactivity was compared between species and sex. Species differences in the proportion of CTR-positive neurons overlapping with CGRP and amylin were observed (Table 3). The percentage of CTR-positive neurons that displayed amylin/CGRP immunoreactivity was significantly higher in humans than in rats. Conversely, the proportion of CTR-positive neurons that did not co-express a peptide was significantly lower for humans than rats (Table 3). The percentage of CTR-positive neurons co-expressing amylin, but not CGRP, was significantly higher for female rats, with a similar trend for female human cases (Table 3).

The percentage of β tubulin III-positive DRG neurons co-expressing CGRP, amylin, and CTR were compared with TG neuron data from previously published data using the same antibodies and image analysis method [14, 17]. Inter-subject variation between human TG cases precluded performing this analysis. Therefore, comparisons were only made between rats and mice. A major difference between DRG and TG is the clear presence of amylin in the DRG. In contrast, a very limited amount of amylin immunoreactivity was observed in rodent TG with Ab254259, which was likely due to a small amount of cross-reactivity with CGRP (Fig. 5A) [17, 28]. Furthermore, the proportion of mouse DRG neurons with CTR was significantly greater than in the TG (Fig. 5A). No other significant differences were observed for mice and rats (Fig. 5A).

## Discussion

### CGRP and amylin are both expressed in C1/2 DRG

This study identified the presence of amylin mRNA and peptide in C1/2 DRG neurons. Amylin-LI exhibited a puncta-like appearance in neuronal cell bodies and a pearl-like appearance in neuronal fibres, suggesting expression in vesicles, neuronal soma and fibres. Based on colocalisation with NF200 and neuronal size, amylin appeared to be present in the cell bodies of C-fibre and A-fibre neurons [8, 36]. This is the first report of amylin and CGRP co-expression in C1/2 DRG neurons, and amylin protein expression in the DRG of humans. Amylin expression in sensory ganglia has been difficult to define, in part due to anti-amylin antibodies frequently detecting CGRP at concentrations estimated to be present in neuronal vesicles [20, 21, 24–29, 39]. Hence, previously reported amylin-LI could represent false positive amylin expression at sites of high CGRP expression [16, 17, 35]. We therefore employed an anti-amylin antibody with limited CGRP cross-reactivity [28, 35]. Furthermore, amylin- and CGRP-LI did not completely overlap, with distinct immunoreactivity in discrete neurons, vesicle-like puncta, and fibres, suggesting true amylin peptide expression. However, higher exposures were required for imaging amylin compared to CGRP, suggesting amylin expression is likely modest, especially when compared to abundant neuropeptides like CGRP. RNA-FISH revealed amylin (*Iapp*) mRNA in rat DRG neurons. These mRNA data do not provide relative levels or the precise location of amylin peptide expression but do suggest that amylin synthesis occurs in the rat DRG [40]. Overall, multiple lines of evidence and consistency between three species indicate that amylin is expressed in upper cervical DRG, where it could play a role in transmitting sensory information, similar to CGRP. This potential physiological role is supported by the use of healthy rodents, rather than disease models.

### Amylin and CGRP in DRG may signal through distinct molecular mechanisms

In addition to amylin and CGRP we report, for the first time, CTR expression in the upper cervical DRG. CTR-LI was present in the DRG of all tested species, using all tested antibodies. There were differences in the proportions of CTR-positive neuronal subtypes between species. In rodents, CTR-LI was predominantly localised in small to medium-sized neuronal cell bodies with low NF200 levels, indicating expression largely in C-fibre neurons [8, 36]. In contrast, neurons exhibiting CTR-LI in human DRG showed greater size variation, suggesting expression in both A- and C-fibre neurons, which is consistent with human single-cell DRG transcriptomics data [41, 42].

We identified that DRG neurons immunopositive for CTR were often (66–84%, Table 3) positive for amylin and/or CGRP. This could have several mechanistic implications. Firstly, both peptides may act as local agonists for CTR-based receptors in the DRG. Secondly, the presence of both ligand and receptor subunit together suggests that CGRP and/or amylin could act via autocrine mechanisms. Few studies have examined the relative distribution of the calcitonin receptor-like receptor (CLR; a component of the “canonical” CGRP receptor which is potently activated by CGRP and more weakly activated by amylin, depending on receptor species) and CGRP in the DRG. However, these studies indicate some co-expression, suggesting that CGRP may also act in an autocrine and paracrine manner at “canonical” CGRP receptors [42, 43]. Therefore, CGRP could mediate biological activity through multiple receptors and signalling mechanisms, whereas amylin could act through CTR-based receptors in a more limited population of neurons.

There are aspects of CGRP biology in sensory neurons and pain that have been difficult to fully explain by the “canonical” CGRP receptor alone. For example, autocrine autoregulatory upregulation of CGRP expression and signalling, a key feature of neuronal sensitisation and pain chronification, is only partially attenuated by CGRP receptor antagonists [9, 11]. Furthermore, CGRP promotes neuronal hyperexcitability and cortical spreading depression [44, 45]. However, fremanezumab (anti-CGRP mAb) and atogepant (CGRP receptor antagonist) cannot effectively inhibit C-fibre neuron activation [45, 46]. This suggests that CGRP could mediate some of its effects, including upregulation of CGRP and the activation and sensitisation of C-fibre neurons, through other receptors, such as the AMY receptors, in addition to the “canonical” CGRP receptor [11, 45, 46]. This aligns with our study, where amylin, CGRP and CTR appeared to be co-expressed in neurons whose size and NF200 expression suggest the C-fibre subtype in rats.

### DRG and TG differentially express CGRP and amylin

The upper cervical DRG and TG innervate the head and have functional and morphological overlap as key sites for mediating craniofacial pain. However, this study, together with previous work, identifies differences in the expression of pain-related neuropeptides between these sensory ganglia [14, 17]. A notable distinction is our observation of higher abundance of amylin in the DRG, compared to the TG. Although human immunofluorescence for TG and DRG was not performed in parallel, we used the same methodology and some matched human cases for TG and DRG, suggesting differences between these cases could be genuine. There were some methodological differences for the rodent data, however, transcriptome data corroborates this difference, ranking amylin as the 8th most differentially expressed gene between rat DRG and TG [47]. Variation in amylin expression between sensory ganglia may not be unexpected as reports suggest differences in amylin expression across DRG levels [20, 21, 24]. Therefore, CTR-based receptors could be activated by two distinct ligands (CGRP and amylin) in the DRG to contribute to pain transmission, in contrast to the TG where only CGRP may be expressed, at least in the absence of disease [14, 15, 17]. Any regulation of amylin has not yet been investigated.

We observed amylin and CGRP immunoreactivity in discrete puncta, indicating different vesicle populations. This suggests there are diverse molecular processes between the DRG and TG involved in transmitting nociceptive information, such as distinct bursts of CGRP and amylin release and/or unique patterns of receptor activation. In addition, the transcription, translation, packaging, and release of amylin and CGRP could be induced under different regulatory mechanisms or temporally controlled, possibly contributing to biologically diverse aspects of pain between these sensory ganglia [48]. The expression of neuropeptides, including CGRP, in dense core vesicles is well characterised, and amylin may be present in a different subset to CGRP [49]. However, other peptides, including the related adrenomedullin, have been reported in clear synaptic vesicles in DRG fibres, which may also be the case for amylin [50]. Further study is needed to identify which vesicle subtypes contain amylin and under which conditions they are formed, regulated, and released.

### Implications for treatment and future directions

Upper cervical DRG nerves, such as the greater occipital nerve, are involved in several craniofacial pain conditions, including occipital and post-traumatic brain injury-associated headaches, and migraine that predominantly affects the back of the head [1, 4, 51, 52]. These sensory neurons are involved in the pain aspects of these disorders, and other symptoms such as aura and neck stiffness [3, 51, 52]. Blocking CGRP alone may not be sufficient for craniofacial pain conditions where the DRG make a substantial contribution because DRG neurons may also express amylin and AMY receptors [3]. For example, migraine pain at the back of the head was four-fold more prevalent in response to the amylin analogue pramlintide than CGRP in a human provocation study, underscoring the potential involvement of AMY receptors in DRG-mediated pain [17]. However, it also possible that amylin and CTR-based receptors may play an anti-nociceptive role because exogenous amylin reduced nociceptive behaviour when administered prior to noxious stimuli, such as formalin and acetic acid [19, 22, 23]. In addition, administration of CTR-based receptor agonists, such as salmon calcitonin, have analgesic properties and are reported to decrease TG and DRG neuron activation [53–57]. This suggests that the contributions that amylin and the CTR-based receptors make to pain and migraine are likely complex and further research into this system is warranted.

Determining which receptors are present and by what mechanisms they contribute to nociceptive signalling and sensitisation is crucial for developing therapeutics. Our signalling data indicate expression of functional CGRP- and amylin-responsive receptors in the DRG. The pharmacology suggests functional CTR expression, potentially as part of AMY receptors, as amylin has relatively limited activity at the rodent CGRP and adrenomedullin receptors, while both CGRP and amylin are equipotent at some rodent AMY receptors [37, 38]. However, the pharmacology of these receptors is complex, and there are some species differences meaning that it is difficult to draw firm conclusions about the role of individual receptors. CLR mRNA and protein has been detected in DRG neurons, therefore, CGRP could potentially signal through CLR and/or CTR-based receptors [42, 43]. Examining RAMP expression in conjunction with CTR and CLR subunits to determine the spatial distribution of receptor-RAMP pairs could help reveal which receptors are relevant. However, many anti-RAMP and anti-CLR antibodies have limitations [33, 58, 59]. The absence of CLR and RAMP immunofluorescence in this study prevents further delineation of the role of each receptor in CGRP and amylin mediated signalling in the DRG. Future studies could consider non-antibody-based methods, such as fluorescent in situ hybridization, mass spectrometry imaging and spatial transcriptomics or proteomics. In addition, the potential for paracrine and autocrine signalling mechanisms should be considered as autocrine signalling is proposed to require higher antagonist concentrations to attenuate receptor activation, relative to paracrine signalling [60].

Our study noted some species and sex differences. For example, we observed a greater co-expression of CTR with amylin, and of peptides in C-fibre neurons in female rats, which may underlie the sex-dependent differences in amylin sensitivity previously reported [17, 18]. We did not use pain models or human cases with a migraine diagnosis. However, previous studies have reported upregulation of CGRP and amylin in the DRG in response to noxious stimuli [20, 21]. In addition, sex-specific and pain-specific differences in expression of the CGRP and amylin peptide and receptor systems in human DRG have been reported [42]. Another consideration is the age of the human cases examined, as the expression of neuropeptides is known to change during ageing [61, 62]. Examination of the spatial relationships of this family of peptides and receptors in younger, and migraine patients will shed additional light on their contribution to nociceptive signalling and potential as therapeutic targets.

## Conclusions

The mechanisms underlying sensory information transmission, sensitisation, and pain chronification remain unclear. This study identified three pain-relevant proteins (CTR, CGRP, and amylin) in the DRG of mice, rats and humans, suggesting a potential role of CTR-based receptors in pain transmission. Given their co-expression profiles, autocrine or paracrine signalling could occur (Fig. 5B). Moreover, amylin might play a greater role in DRG-mediated pain than the related sensory ganglia (TG). Developing drugs targeting amylin and the AMY receptors could benefit patients with DRG-mediated pain disorders or respond poorly to existing anti-CGRP pathway therapies but further work is needed to test these hypotheses.

### Supplementary Information

**Supplementary Material 1.**

## Data Availability

The datasets used and/or analysed during the current study are available from the corresponding author on reasonable request.

## Abbreviations

CGRP: calcitonin gene-related peptide
AMY receptor: amylin receptor
AMY_1_: amylin 1 receptor
GPCR: G protein-coupled receptor
DRG: dorsal root ganglia
TG: trigeminal ganglia
CLR: calcitonin receptor-like receptor
CTR: calcitonin receptor
SD: Sprague Dawley
PBS: phosphate buffered saline
TBS: tris buffered saline
RNA-FISH: RNA-fluorescent in situ hybridization
mRNA: messenger ribonucleic acid
