# Supplementary material for: Calcitonin receptor, calcitonin gene-related peptide and amylin distribution in C1/2 dorsal root ganglia
Source: J Headache Pain. 2024 Mar 14;25(1):36. doi: 10.1186/s10194-024-01744-z (PMC10938748; doi:10.1186/s10194-024-01744-z)
Supplement: Supplementary file 1 — Supplementary Material 1. [file 10194_2024_1744_MOESM1_ESM.docx]

**Calcitonin receptor, calcitonin gene-related peptide and amylin distribution in C1/2 dorsal root ganglia**

Tayla A. Rees^1,2^, Zoe Tasma^1,2^, Michael L. Garelja^2,3^, Simon J. O’Carroll^4^, Christopher S. Walker^1,2^, Debbie L. Hay^2,3^

^1^School of Biological Sciences, University of Auckland, Auckland, New Zealand

^2^Maurice Wilkins Centre for Molecular Biodiscovery, University of Auckland, Auckland, New Zealand

^3^Department of Pharmacology and Toxicology, University of Otago, Dunedin, New Zealand

^4^Department of Anatomy and Medical Imaging and Centre for Brain Research, Faculty of Medical and Health Science, University of Auckland, Auckland, New Zealand

**Supplementary Methods**

**Rodent origins and care:**

Male and female Sprague Dawley (SD) rat and C57BL/6J mouse DRG for **Figures 1-3, 4C** and **E** were obtained from the Integrated Physiology Unit (University of Auckland, Auckland, New Zealand). All procedures involving the use of animals were conducted in accordance with the New Zealand Animal Welfare Act (1999) and approved by the University of Auckland Animal Ethics Committee. Rodents of the same sex were housed with littermates in Techniplast Greenline IVC with Sealsafe Plus GM500 cages (mice) or as pairs in Techniplast Conventional 1500U cages (rats) in a controlled environment (12 h light–dark cycle; room temperature, 22 ± 2 ^o^C) with ad libitum access to standard chow (Teklad TB 2018; Harlan, Madison, WI, USA) and water. Cages also contained an additional enrichment item (house or toy). The oestrous cycle phase was not assessed or recorded for female rats or mice. Animals were euthanized by CO_2_ inhalation and cervical dislocation as part of routine colony maintenance.

Neonatal Wistar rats for **Figure 2F** were also obtained from the Integrated Physiology Unit (University of Auckland) in accordance with the New Zealand Animal Welfare Act (1999) and approved by the University of Auckland Animal Ethics Committee. 16 rat pups (3-5 days old) were used for this study. Neonatal animals were euthanized by decapitation with surgical scissors. At least two pups remained with the dam to reduce stress and complications. Male and female rat pups were not distinguished and therefore, no comparisons between sex were made for cAMP assays.

Male and female Sprague Dawley (SD) rat DRG for **Figure 4A** were obtained from the Biomedical Research Facility (University of Otago, Dunedin, New Zealand). All procedures involving the use of animals were conducted in accordance with the New Zealand Animal Welfare Act (1999) and approved by the University of Otago Animal Ethics Committee. Up to three rats of the same sex were housed with littermates in individually ventilated cages in a controlled environment (12 h light-dark cycle; room temperature 22 ± 2 ^o^C) with ad libitum access to irradiated standard chow (Teklad TB 2918). Cages also contained an additional enrichment item (house or toy). The oestrous cycle phase was not assessed or recorded for female rats. Animals were euthanized by pentobarbital overdose (100 – 150 mg/kg, delivered intraperitoneally). Upon absence of paw withdrawal reflexes, rats were decapitated using a custom-built guillotine.

Rat and mouse information (weight, sex, age) is detailed in **Table S2**; animals from the University of Auckland are identified by numbers, while animals from the University of Otago are identified by letters.

**Human DRG details and processing:**

Postmortem human DRG were obtained from the University of Auckland Human Anatomy Laboratory, with informed consent by the donor before death and next of kin after death as part of the University of Auckland Human Body Bequest Program for teaching and research. This program and its procedures operate under the Human Tissue Act of 2008 and are overseen by the New Zealand Police Inspector of Anatomy. Human case information (sex, age, cause of death) is detailed in **Table S3**.

After dissection from the cadaver, DRG specimens were fixed with 15% formaldehyde in 0.1 M phosphate buffer for 24 h at 4 °C. Specimens were then dehydrated in sequential incubations of 70%, 80%, 95%, and 100% ethanol, followed by clearing with xylene as per a standard, pre-set ‘biopsy’ cycle in a tissue processor (ASP6025, Leica Biosystems) at RT under vacuum. Specimens were then embedded in paraffin wax and sectioned sagittally (10 μm) on a rotary microtome (Leica Biosystems, HI 2235). Sections were floated in a water bath set at 37 °C (Leica Biosystems, HI1210), mounted individually on SuperFrost slides, and allowed to dry at RT for at least 18 h before storage at RT indefinitely.

**Image analysis:**

Image analysis was performed on multiple fields of view (20x high NA lens, Operetta) on each section from individual animals; two-to-three fields of view for mice, four for rats, and five for humans. Each field of view contained 30-160 neurons. All images were analysed as unedited 16-bit TIFFs in greyscale. Image analysis was performed using FIJI and was partially automated using the macro function to generate counts and the mean diameter (mean of Feret diameter and minimum diameter for each neuron) of neurons.

***Determining the threshold intensity for “positive” CGRP, amylin, or CTR neurons:***

Prior to analysis of the images the minimum threshold for CGRP, amylin, and CTR immunoreactivity was determined for each mouse, rat, or human case. Due to the variability in staining patterns and intensity (e.g. for CGRP this ranged from intense cytoplasmic staining to puncta), global thresholding could not be applied for amylin, CGRP, and CTR. Therefore, a minimum threshold was determined for each animal by analysing the fluorescence intensity of 20-30 positive and negative neurons in two fields of view for each rat, mouse, or human case. The intensity of the positive and negative neurons were statistically summarized (descriptive statistics) using Prism GraphPad 8.0.2 and the 10^th^ percentile value of the positive staining was used as the minimum threshold for that particular rat, mouse, or human case in the image analysis. To confirm that this value would not result in the inclusion of non-specific background staining this was compared to the 90^th^ percentile intensity value for negative neurons. In all cases the 10^th^ percentile value of positive neurons and 90^th^ percentile value of negative neurons did not overlap, suggesting that any staining selected for by this thresholding method would likely be capturing positive staining above background.

***Proportion of DRG neurons (β tubulin III) that express CGRP, amylin, or CTR:***

The proportions of DRG neurons that expressed CGRP, amylin, and CTR were quantified using sections co-stained with β tubulin III. First, the β tubulin III image was thresholded using the triangle global threshold, set to automatic, and a binary mask created. The shape filter plugin was applied (area: 20000 for rat/mouse, 250 for human; fill holes) to remove some of the fibre staining. The neuronal cell bodies for rat and human were then separated by adjustable watershed (1.5) and another shape filter was used (area: 20000 for rat, 250 for human) to remove debris and fibre staining. Unfortunately, as the mouse DRG neurons were very densely packed the adjustable watershed was not able to segment the β tubulin III sufficiently, resulting in neurons close together being counted as one large neuron affecting the ability to accurately determine the number and size of the mouse neurons. Therefore, in this case the β tubulin III mask was not segmented but was kept for overlay with the CGRP, amylin and CTR masks, as it represented the regions in the picture where β tubulin III is expressed, and the number of neurons was manually counted instead.

Next, CGRP and amylin or CTR images were analysed by thresholding with the predetermined appropriate minimum threshold for each animal or human case. These images were similarly converted to binary masks and underwent a shape filter (area: 20000 for rat/mouse, 250 for human; fill holes), adjustable watershed and another shape filter (area: 20000 for rat/mouse, 250 for human) to remove debris, non-specific speckles and fibre staining. To overlay the β tubulin III mask with the amylin, CGRP, or CTR masks, morphological reconstruction was used (MorphoLib plugin). The β tubulin III/CGRP and β tubulin III/amylin or β tubulin III/CTR masks were then overlayed with MorphoLib to generate a CGRP/amylin or CGRP/CTR mask to show neurons which only co-express CGRP and amylin or CTR. The properties of the three overlayed masks were then adjusted with the correct pixel width/height for the 20x high NA Operetta lens. The area and Feret diameter were determined for each individual neuron using “Analyze Particles” and were also summarized with counts for β tubulin III neurons, CGRP+ neurons, amylin+ neurons, amylin/CGRP+ neurons, CTR+ neurons, and CTR/CGRP+ neurons. During method development, summarized counts and diameters were compared to manual counting and measurement for a subset of animals as a quality control and validation of the analysis.

***Proportion of CGRP, amylin, or CTR positive neurons which co-express NF200:***

Similar to the above analysis, using rat DRG sections co-stained with NF200, CGRP and amylin or NF200, CGRP and CTR. First, the NF200 image was thresholded using the triangle global threshold, set to automatic, and a binary mask created. The shape filter plugin was applied (area: 20000; fill holes), neuronal cell bodies segmented by adjustable watershed (1.5), and shape filter applied again (area: 20000). The CGRP, amylin, and CTR binary masks were generated as described above, overlayed and analysed in the same manner. The binary masks for CGRP, amylin, CTR, CGRP/amylin, and CGRP/CTR were analysed for “neuron” number with “Analyze Particles” before (1) and after (2) overlay with NF200 to determine the total number of neurons expressing the protein/s of interest (1) and the number of numbers of neurons expressing the protein/s and NF200 (2).

Due to the compact and densely packed nature of the neurons in mouse DRG it is difficult to properly segment individual cells. As a consequence, neurons with low NF200 staining close to neurons with intense NF200 staining would be included in the analysis, leading to false positives. Therefore, quantification of the co-expression of CTR, CGRP, and amylin with NF200 could not be performed for mouse DRG.

***Proportion of CTR neurons which co-express CGRP, amylin, or CGRP and amylin:***

Analysis was performed on sections co-stained with CTR, CGRP, and amylin. A mouse anti-CTR is required to co-stain with the goat anti-CGRP and rabbit anti-amylin, therefore, this could not be performed in mice due the high background staining generated by the donkey anti-mouse secondary antibodies. CGRP and amylin or CTR images were thresholded with the predetermined appropriate minimum threshold for each animal or human case. These images were similarly converted to binary masks and underwent a shape filter (area: 20000 for rat/mouse, 250 for human; fill holes), adjustable watershed and another shape filter (area: 20000 for rat/mouse, 250 for human). Morphological reconstruction (MorphoLib plugin) was used to overlay the CTR mask with the CGRP or amylin to identify CTR+ cells which also co-express CGRP (CTR/CGRP) or amylin (CTR/amylin). The CTR/CGRP and CTR/amylin masks were also overlayed to identify CTR+ cells which co-express CGRP and amylin (CTR/CGRP/amylin). The binary masks for CTR, CTR/CGRP, CTR/amylin and CTR/CGRP/amylin were analysed for “neuron” number, area and ferret diameter with “Analyze Particles”.

**Data and statistical analysis for image analysis:** For each section/animal/human case, neuronal cell body diameter data from all fields of view were centre binned in 2.5 μm increments. These data were then plotted to generate histograms to visualize the combined data from each species, presented as mean ± standard error of the mean (s.e.m.) from six individual animals or four human cases. A cubic spline was applied to help visualize the distribution. For each section/animal/human case, the proportion (percentage) of neurons expressing amylin, CGRP, or CTR individually or together was determined for each field of view, three for mice and rats and five for human cases. These were combined to give a mean percentage value for each animal/human case. The mean percentage values were combined for each sex and species as appropriate and presented as mean ± s.e.m from three (sex) or six (species) individual animals (mice/rats) and two (sex) or four (species) for human cases. The combined mean values from three (sex) individual animals were compared using two-tailed unpaired Student’s *t*-tests, or four/six individual animals or human cases (species) were compared using one-way ANOVA with post hoc Bonferroni test. Statistical significance was defined as *p<0.05*.

**Image processing for presentation of IHC and RNA-FISH results:** Representative immunohistochemistry and RNA-FISH images are presented from at least three independent experiments performed using separate antibody or primer/hairpin dilutions. RNA-FISH images were processed with the same set intensity across the images from the same experiment to allow for direct comparisons between the conditions.

Images were minimally processed using the FIJI open-source imaging platform to adjust colour and brightness for presentation purposes ^1^. Any processing was uniformly applied across each image and all conditions for an antibody. Adjustment of contrast and brightness was made via the histogram (contrast stretching) to enhance visualization of positively stained cells with varying intensities, in line with image presentation and data visualization guidelines ^2^. The IHC images are representative images to allow visualization of the immunoreactivity which was previously quantified using raw unmodified TIFFs.

**Dot Blotting:** Dot blotting was performed as previously described ^3^. Briefly, stock solutions of rat/mouse αCGRP and mouse βCGRP peptides were serially diluted in sterile water to give the required concentrations. The peptide order was randomized. Two microliters containing the total amount of each peptide required was loaded as a single spot on 0.45 μm nitrocellulose membranes (Bio-Rad, Hercules, CA). Membranes were then incubated for 1 h at RT in TBS-T with 5% (w/v) low-fat milk (assay buffer). This buffer was removed, and the membranes were then incubated with primary anti-CGRP antibody ab36001 (Supplementary Table S1) diluted 1:1000 in assay buffer for 1 h at RT. Membranes were then washed twice for 5 min in TBS-T and incubated with secondary antibodies diluted 1:1,000 in assay buffer for 1 h at RT. Membranes were then washed twice before the blots were developed with SuperSignal West Pico PLUS (34577, ThermoFisher Scientific) for ~5 min. Blots were imaged using an Amersham A600 imager (GE Healthcare, Chicago, IL). Image acquisition was performed using the automated exposure function with the high dynamic range setting. Blots presented are representative of consistent results from at least two independent experiments.

**Supplementary Tables:**

**Table S1: Details of the antibodies and plasmids used in this study**

| **Antibodies** | | | | | | | |
| --- | --- | --- | --- | --- | --- | --- | --- |
| **Catalogue number and supplier** | **RRID** | **Antigen** | | **Species raised in** | **Poly or monoclonal** | **Dilution, final µg/ml** | **Secondary** |
| **Mouse DRG histology** | | | | | | | |
| 188, Welcome Receptor Antibodies Pty | AB_297696 | Rat CTR C-terminal  GLPIYICHQEPRNPPVSNN | | Rabbit | Polyclonal | 1:500, 20 µg/ml | 1:200 Donkey anti-rabbit AF647  (A31573, ThermoFisher, AB_2536183) |
| Ab36001, Abcam | AB_725807 | Rat αCGRP 22-37 | | Goat | Polyclonal | 1:500, 10 µg/ml | 1:200 Donkey anti-goat AF555 (A21432, ThermoFisher, AB_2535853) |
| Ab254259, Abcam | None available | Mouse amylin 13-37 | | Rabbit | Monoclonal | 1:100, 5.47 µg/ml | 1:200 Donkey anti-rabbit AF647 (A31573, ThermoFisher, AB_2536183) |
| T8578, Sigma | AB_1841228 | β tubulin III | | Mouse | Monoclonal | 1:500, 2 µg/ml | 1:200 Donkey anti-mouse AF488  (A32766, ThermoFisher, AB_2762823) |
| N0142, Sigma | AB_477257 | NF200 | | Mouse | Monoclonal | 1:200, ~3 µg/ml | 1:200 Donkey anti-mouse AF488 (A32766, ThermoFisher, AB_2762823) |
| **Rat DRG histology** | | | | | | | |
| 188, Welcome Receptor Antibodies Pty | AB_297696 | Rat CTR C-terminal  GLPIYICHQEPRNPPVSNN | | Rabbit | Polyclonal | 1:1000, 10 µg/ml | 1:200 Donkey anti-rabbit AF647 (A31573, ThermoFisher, AB_2536183) |
| 8B9, Welcome Receptor Antibodies Pty | AB_2891126 | Rat CTR C-terminal  GLPIYICHQEPRNPPVSNN | | Mouse | Monoclonal | 1:50, 20 µg/ml | 1:200 Donkey anti-mouse AF647  (A31571, ThermoFisher, AB_162542) |
| Ab36001, Abcam | AB_725807 | Rat αCGRP 22-37 | | Goat | Polyclonal | 1:500, 10 µg/ml | 1:200 Donkey anti-goat AF555 (A21432, ThermoFisher, AB_2535853)  1:200 Donkey anti-goat AF488  (A11055, ThermoFisher, AB_2534102) |
| Ab254259, Abcam | None available | Mouse amylin 13-37 | | Rabbit | Monoclonal | 1:100, 5.47 µg/ml | 1:200 Donkey anti-rabbit AF647 (A31573, ThermoFisher, AB_2536183)  1:200 Donkey anti-rabbit AF555  (A31572, ThermoFisher, AB_162543) |
| T8578, Sigma | AB_1841228 | β tubulin III | | Mouse | Monoclonal | 1:500, 2 µg/ml | 1:200 Donkey anti-mouse AF488 (A32766, ThermoFisher, AB_2762823) |
| N0142, Sigma | AB_477257 | NF200 | | Mouse | Monoclonal | 1:200, ~3 µg/ml | 1:200 Donkey anti-mouse AF488 (A32766, ThermoFisher, AB_2762823) |
| **Human DRG histology** | | | | | | | |
| 31-01, Welcome Receptor Antibodies Pty | AB_530749 | Human CTR C-terminal  DIPIYICHQEPRNEPANN | | Mouse | Monoclonal | 1:200, 5 µg/ml | 1:200 Donkey anti-mouse AF647 (A31571, ThermoFisher, AB_162542)  1:200 Donkey anti-mouse AF594  (A21203, ThermoFisher, AB_141633) |
| Ab36001, Abcam | AB_725807 | Rat αCGRP 22-37 | | Goat | Polyclonal | 1:500, 10 µg/ml | 1:200 Donkey anti-goat AF555 (A21432, ThermoFisher, AB_2535853) |
| Ab254259, Abcam | None available | Mouse amylin 13-37 | | Rabbit | Monoclonal | 1:100, 5.47 µg/ml | 1:200 Donkey anti-rabbit AF647 (A31573, ThermoFisher, AB_2536183) |
| T8578, Sigma | AB_1841228 | β tubulin III | | Mouse | Monoclonal | 1:500, 2 µg/ml | 1:200 Donkey anti-mouse AF488 (A32766, ThermoFisher, AB_2762823) |
| T2200, Sigma | AB_262133 | β tubulin III | | Rabbit | Monoclonal | 1:500, 1.2 µg/ml | 1:200 Donkey anti-rabbit AF488 (A21206, ThermoFisher, AB_2535792) |
| **Plasmids** | | | | | | | |
| **Catalogue number and supplier** | | **Gene** | **Species** | **Reference sequence (mRNA)** | | **Vector** | |
| Custom synthesis, Gene Universal | | *Iapp* | Rat | NM_012586.1 | | pcDNA3.1+ | |
| Custom synthesis, Gene Universal | | *Calca* | Rat | NM_001033955.1 | | pcDNA3.1+ | |
| Custom synthesis, Gene Universal | | *Calcb* | Rat | NM_138513.2 | | pcDNA3.1+ | |

**Table S2: Rodent details**

| **Rats** | | | | **Mice** | | | |
| --- | --- | --- | --- | --- | --- | --- | --- |
| **Animal** | **Sex** | **Weight (g)** | **Age (weeks)** | **Animal** | **Sex** | **Weight (g)** | **Age (weeks)** |
| Rat 1 | Female | 285 | 16 | Mouse 1 | Male | 27.6 | 14 |
| Rat 2 | Female | 269 | 16 | Mouse 2 | Male | 27.6 | 14 |
| Rat 3 | Male | 575 | 21 | Mouse 3 | Female | 21.8 | 11 |
| Rat 4 | Male | 607 | 21 | Mouse 4 | Female | 21.8 | 11 |
| Rat 5 | Female | 320 | 31 | Mouse 5 | Female | 22.6 | 16 |
| Rat 6 | Male | 710 | 31 | Mouse 6 | Male | 32.7 | 16 |
| Rat 7 | Female | 353 | 21 |  | | | |
| Rat 8 | Female | 374 | 21 |  |  |  |  |
| Rat 9 | Male | 575 | 15 |  |  |  |  |
| Rat A | Female | 220 | 8 |  |  |  |  |
| Rat B | Female | 222 | 8 |  |  |  |  |
| Rat C | Male | 305 | 8 |  |  |  |  |
|  | | | | | | | |

**Table S3: Human case details**

| **Case** | **Sex** | **Age (years)** | **Cause of death** |
| --- | --- | --- | --- |
| A1201 | M | 94 | Bronchopneumonia |
| A1228 | M | 65 | Hypoxia |
| A1246 | F | 99 | Bronchopneumonia |
| A1266 | F | 89 | Inanition |

**Supplementary Results**


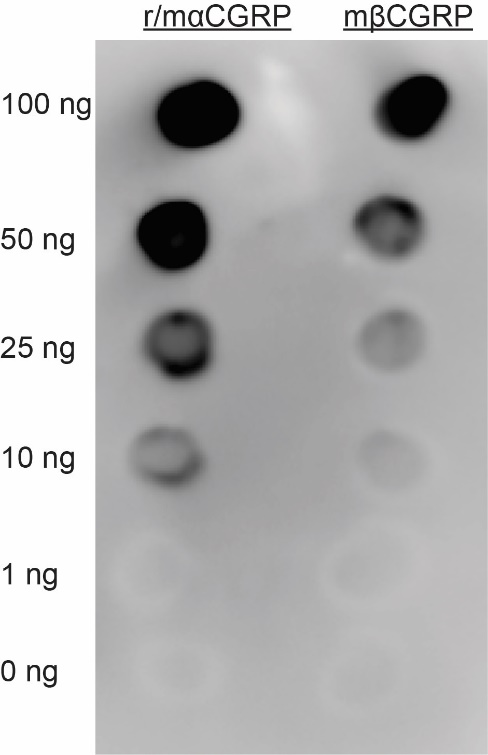


**Figure S1: Immunoblots using full-length rat/mouse αCGRP and mouse βCGRP and anti-CGRP antibody Ab36001 (1:1000) with varying quantities of each peptide.** Image is representative of two independent experiments. Brightness and contrast have been adjusted for presentation purposes. CGRP, calcitonin gene-related peptide.


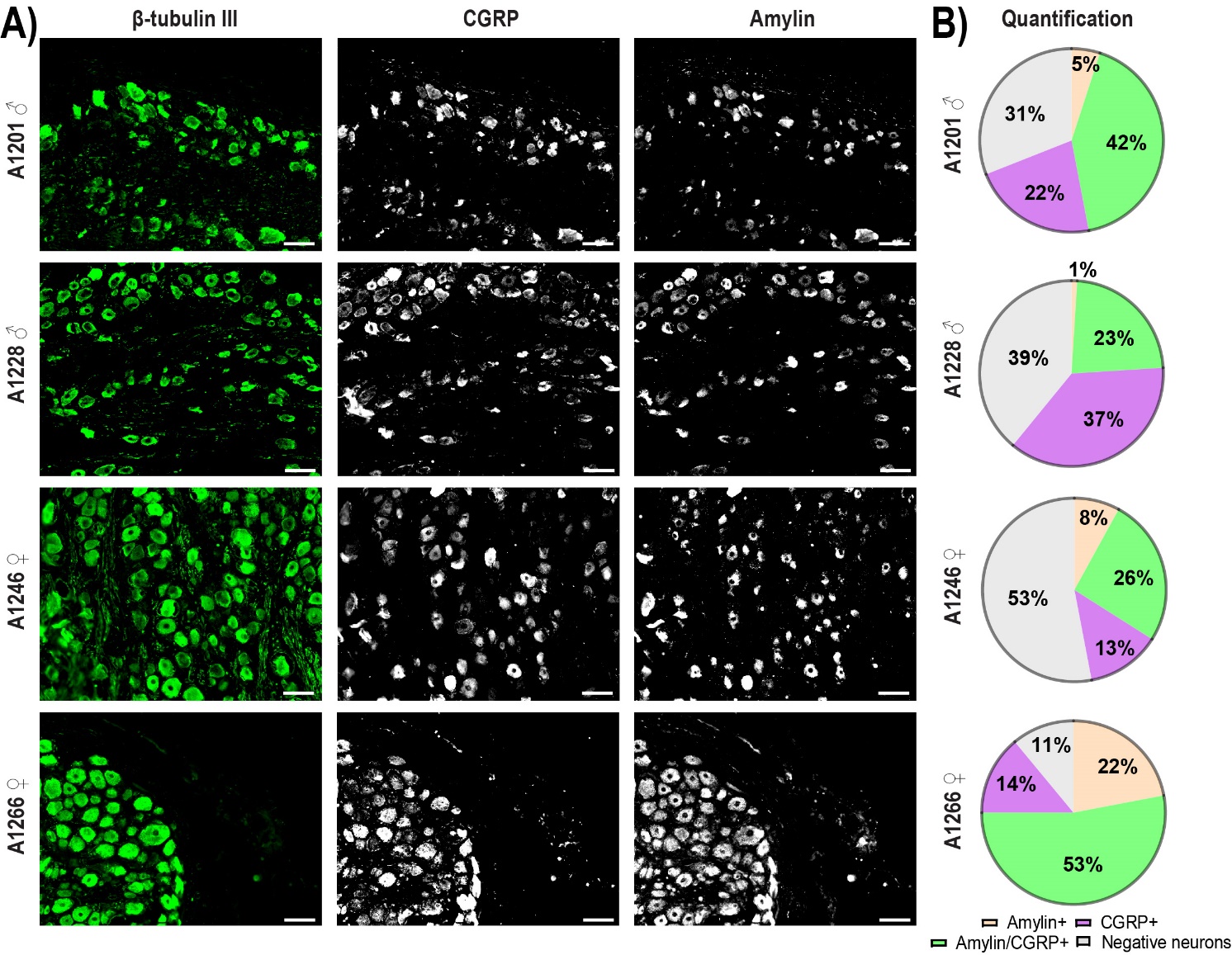


**Figure S2: Variation in CGRP and amylin immunoreactivity across the four human cases.** A) CGRP and amylin immunoreactivity in DRG neurons (β tubulin III) for the different human cases. Brightness and contrast have been adjusted for presentation purposes and consistently applied across the four cases. Scale bar, 100 µm. Images are representative of at least five fields of view per case and two technical replicates. B) Quantification of the percentage of the human DRG neuronal population (β tubulin III) which express amylin alone (orange), CGRP alone (purple), or co-express amylin and CGRP together (green) for each human case. Negative neurons (grey) refer to the population of neurons (β tubulin III) which do not express amylin or CGRP. Data are mean of five fields of view per human case.


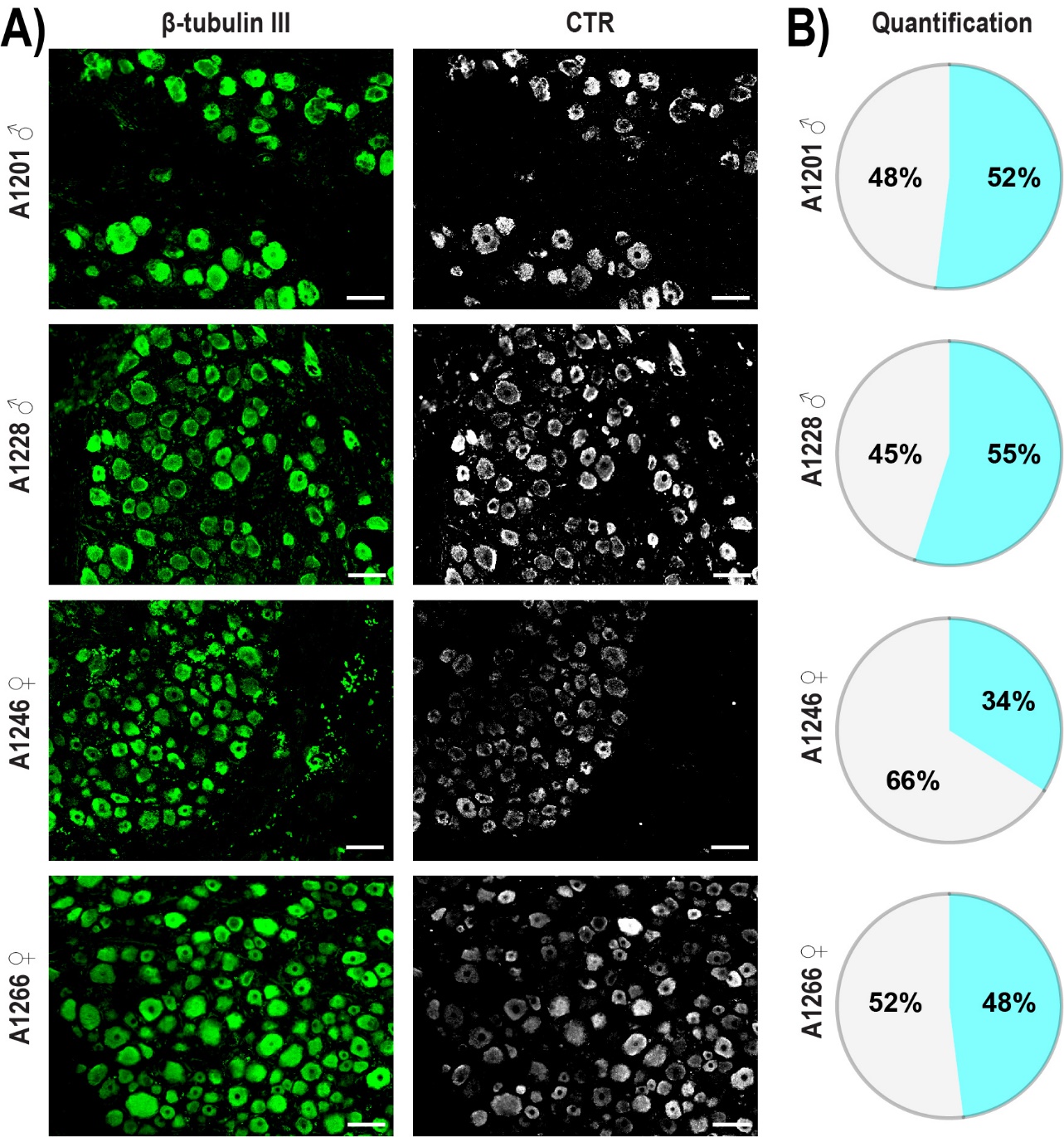


**Figure S3: Variation in CTR immunoreactivity across the four human cases.** A) CTR immunoreactivity in DRG neurons (β tubulin III) for the different human cases. Brightness and contrast have been adjusted for presentation purposes and consistently applied across the four cases. Scale bar, 100 µm. Images are representative of at least five fields of view per case and two technical replicates. B) Quantification of the percentage of the human DRG neuronal population (β tubulin III) which express CTR (blue) for each human case. Negative neurons (grey) refer to the population of neurons (β tubulin III) which do not express CTR. Data are mean of five fields of view per human case.


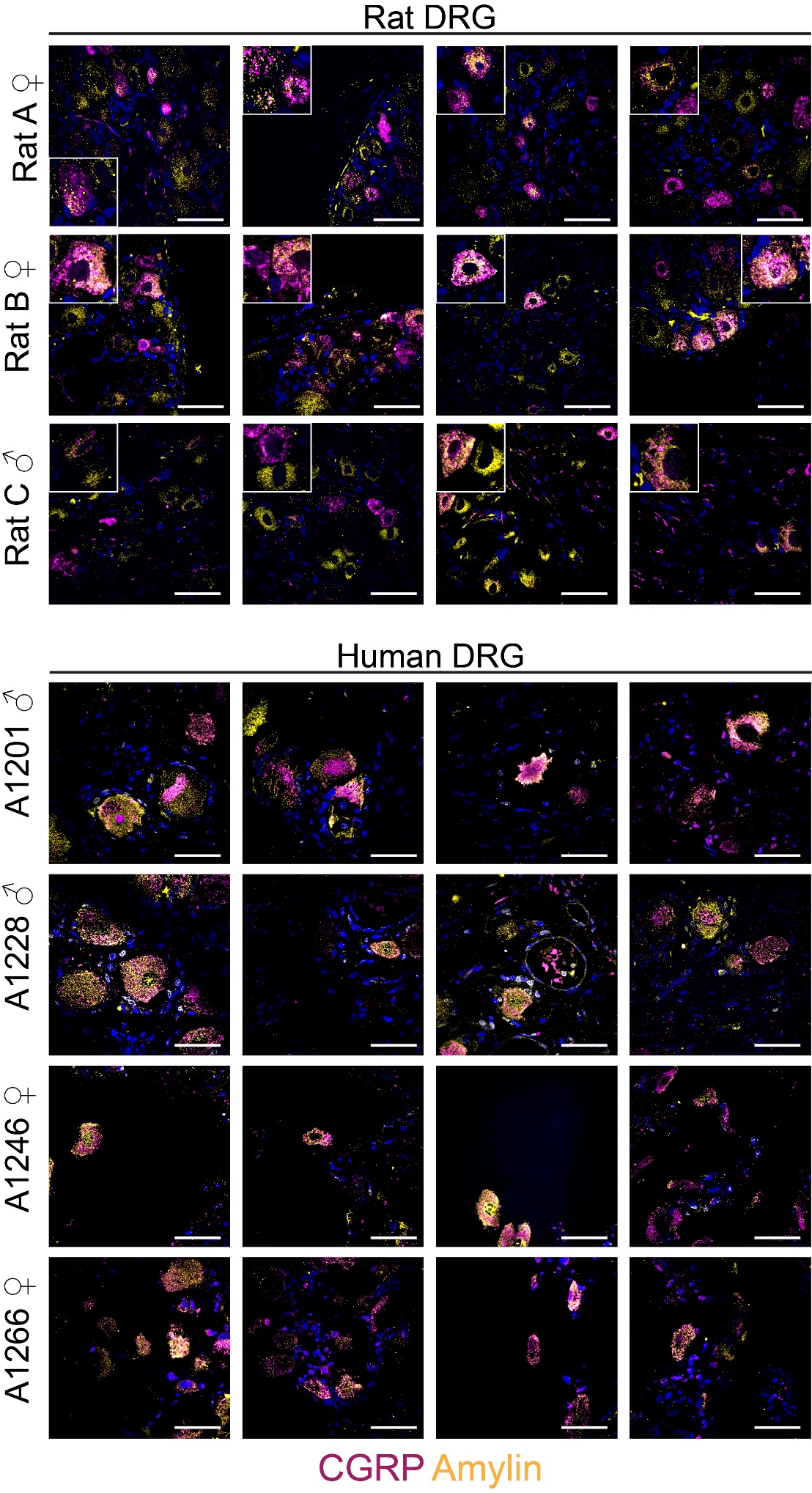


**Figure S4: CGRP and amylin are expressed in distinct vesicles in rat and human DRG neurons.** CGRP immunoreactivity in purple, amylin immunoreactivity in yellow. DAPI staining of nuclei in blue. Four fields of view at 63x of each rat or human case. Image brightness and contrast were adjusted for presentation purposes and merged in FIJI. Scale bar, 50 µm. Images are representative for each of the rats and human cases shown.


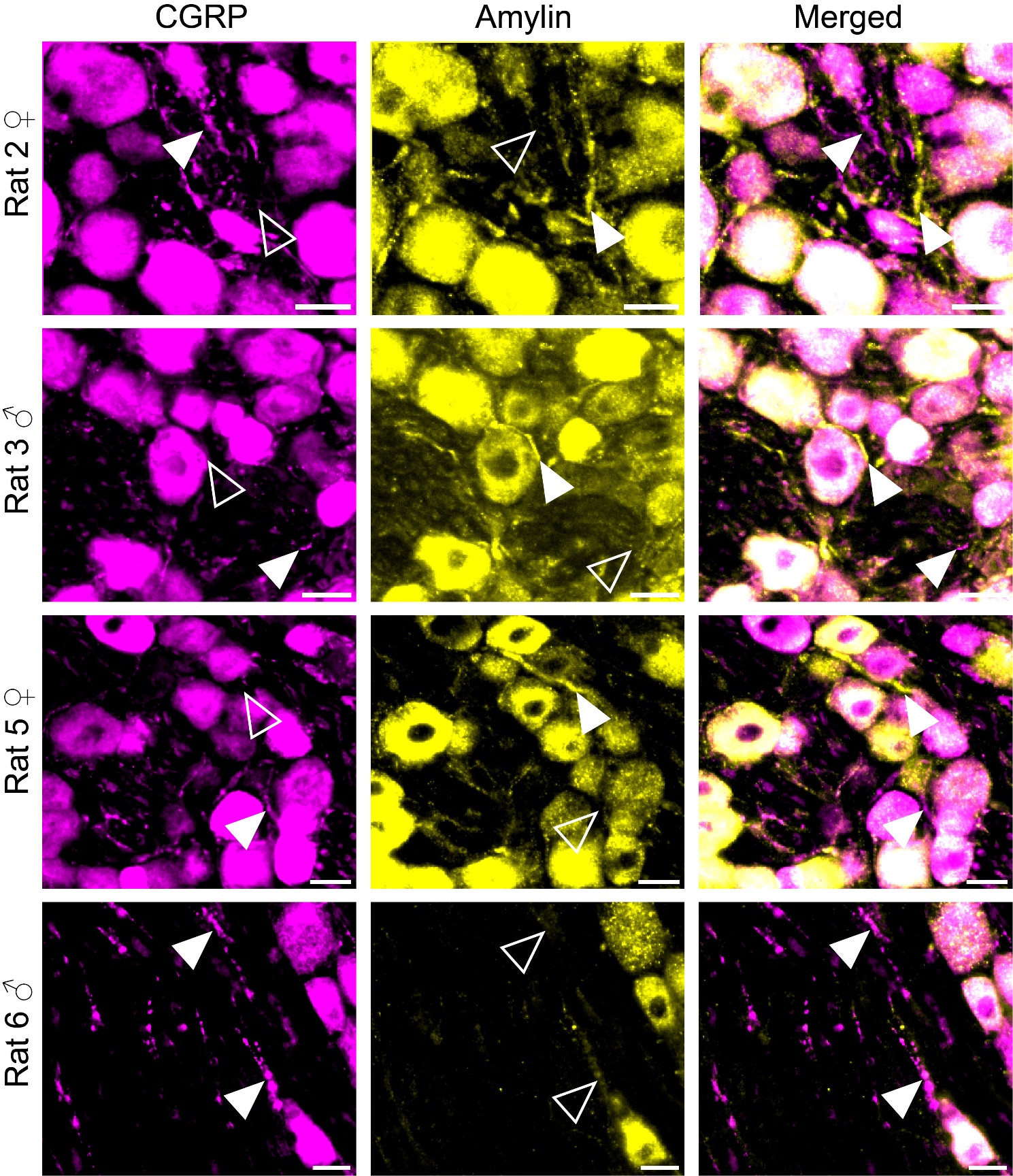


**Figure S5: CGRP and amylin are expressed in rat DRG fibres.** Arrows indicate examples of fibres which express one peptide but not the other. Filled white arrowheads indicate examples of positive immunoreactivity; empty arrowheads indicate examples of an absence of immunoreactivity. Image brightness and contrast were adjusted for presentation purposes and merged in FIJI. Scale bar, 20 µm. Images are representative for each of the rats shown.

**References:**

1. Schindelin J, Arganda-Carreras I, Frise E, et al. Fiji: an open-source platform for biological-image analysis. Nat Methods. 2012 Jun 28;9(7):676-82.

2. Johnson J. Not seeing is not believing: improving the visibility of your fluorescence images. Mol Biol Cell. 2012 Mar;23(5):754-7.

3. Rees TA, Hay DL, Walker CS. Amylin antibodies frequently display cross-reactivity with CGRP: characterization of eight amylin antibodies. Am J Physiol Regul Integr Comp Physiol. 2021 Feb 10;320(5):R697-R703.
